# Environmental volatility is associated with adaptive caution in visual search

**DOI:** 10.1371/journal.pcbi.1014310

**Published:** 2026-10-21

**Authors:** Nan Qiu, Fredrik Allenmark, Siyi Chen, Hermann J. Müller, Zhuanghua Shi

**Affiliations:** 1 The Clinical Hospital of Chengdu Brain Science Institute, MOE Key Lab for Neuroinformation, University of Electronic Science and Technology of China, Chengdu, China; 2 Neuro-cognitive Psychology, Department of Psychology, LMU Munich, Munich, Germany; University of Birmingham, UNITED KINGDOM OF GREAT BRITAIN AND NORTHERN IRELAND

## Abstract

Real-world distractors occur in environments whose states change at different rates. We asked whether such volatility alters initial overt oculomotor allocation, later target verification and response commitment, or both. Observers performed an additional-singleton search task with concurrent eye tracking while distractor presence followed high- or low-volatility sequences, with overall distractor prevalence held constant. Trial-pooled oculomotor capture was higher under high volatility, a pattern that appears to indicate altered filtering. Matching the same-location run position removed that difference, with no detectable difference in capture across volatility regimes at matched run position. The pooled capture effect was therefore consistent with a structural consequence of the volatility manipulation, which enriched high-volatility blocks with early-run positions where capture is intrinsically high. The volatility effect itself appeared on distractor-absent trials. Under high volatility, response times were longer, fixations more frequent, final-target dwell longer, and errors fewer, while target-acquisition latency itself was unchanged. Same-location repetition learning showed no detectable difference in slope across regimes. A hierarchical drift-diffusion model (DDM) and a complementary volatility Kalman-filter (VKF) comparison converged in showing that manual responses carried both boundary-related and drift-related structure. With distractor frequency held constant, the per-trial effect of volatility therefore emerged after the first saccade as an increase in response caution rather than in oculomotor capture. This pattern is consistent with precision-weighted adjustment to environmental uncertainty.

## Author summary

Visual environments differ not only in how often distracting objects appear but also in how frequently their presence changes. We asked whether this volatility changes what first captures the eyes or how carefully people inspect a scene before they respond. Participants searched for a tilted target while an irrelevant tilted distractor appeared in either rapidly changing or more stable sequences, with its frequency held constant. In the rapidly changing sequence, the first eye movement was directed toward the distractor more often. However, this difference followed from the sequences themselves because rapid change produced more trials on which the distractor had moved to a new location, where it was most likely to attract the first eye movement. Once we compared trials with the same repetition history, the first eye movement no longer differed between the two environments. The volatility effect appeared later, on trials without a distractor. In the rapidly changing environment, participants made more saccades, inspected the target for longer, responded more slowly, and made fewer errors. Computational models pointed the same way: people accumulated evidence more slowly and required more of it before committing. Volatility thus made observers more cautious without measurably changing what first captured their gaze.

## Introduction

A patch of sunshine in March tells you little about the next hour, but a sunny afternoon in July almost certainly extends into the evening. The two skies can contain the same proportion of sunshine and still differ in how often the weather changes. The rate is what cognitive scientists call *volatility*: how often the environment’s hidden state changes, independently of how often any one state occurs [1,2]. People track volatility and use it to decide how much weight new information deserves. In an unstable environment, older experience becomes less reliable and new observations gain weight for prediction. In a stable environment, a surprising event is more likely to be noise and is discounted against the accumulated evidence [1,3]. Formally, this is a precision-weighting operation that sets the gain on recent evidence relative to accumulated prior belief [2–6]. Whether the same logic extends to one of the most basic visual search operations, the suppression of salient distractors, is only beginning to be examined.

Distractor suppression is known to depend on experience. Observers learn where and when distractors tend to appear and down-weight the impact of predictable ones, lowering their priority on an attention-guiding spatial map [7–10], and when distractor and target differ in feature dimension, filtering the irrelevant channel outright [11,12]. If distractor suppression is learned, it should depend not only on how often distractors occur but also how often that pattern changes, and the regularities imposed over different timescales do modulate it [13,14]. What those studies do not isolate is volatility itself: the switching rate of distractor presence versus absence, with distractor prevalence held constant. Our earlier work isolated volatility using search displays with a long-term distractor hotspot, where one region contained the distractor far more often than any other location, and showed that volatility modulated how strongly observers deployed the learned spatial distractor prior [15]. Such a design provides a durable spatial regularity, so it can only show volatility modulating a prior that is already in place. The question is what happens when no such spatial prior exists, and the only learnable regularity is the trial-to-trial repetition of the distractor’s state and location. In the present study, we therefore removed the distractor hotspot by rendering every non-target location equally likely to contain the distractor. So, suppression had to be established online, from short-term repetition alone.

Answering this question requires specifying what exactly volatility might change, because suppressing a distractor involves several processes. A volatile environment could alter the gain on an early distractor signal, the time needed to disengage once gaze has landed on the distractor, and/or the criterion for committing to a response once the target has been found. These are different processes with different signatures, yet the standard reaction-time interference index (distractor-present minus distractor-absent RT) compresses all of them into a single number, in which effects arising at different stages can even cancel [16,17]. However, eye movements can resolve this sequence into its stages. The first saccade is itself a decision: its destination records which location won the earliest competition among stimulus salience, task set, learned priority, and saccade planning [18–20]. The subsequent measures index processing after this initial allocation: the distractor dwell time, target acquisition and verification, and finally the manual response and its accuracy [21–23]. None of these measures isolates the distraction process completely. Together, they separate initial gaze allocation from later search and response stages, helping to characterize which stages of visual search are influenced by volatility.

We manipulated volatility between two within-subject sessions (the high- and low-volatility regimes) by varying the probability that the distractor-present or -absent state persisted from one trial to the next. Overall distractor prevalence was held at 50%, and eye movements were recorded throughout. Within a session, a *run* is a consecutive stretch of trials sharing the same distractor state, and a trial's *run position* is its ordinal place in that stretch. Because the distractor occupies a single location for the length of a present run, run position also counts how many times that location has repeated. The persistence probability determines both the state-change rate and run length. High volatility produces shorter runs and thus mostly earlier run positions, whereas low volatility produces longer runs and later run positions. This difference matters because distractor handling depends on recent history. Interference is reduced when a distractor repeats at the location it occupied on the preceding trial [8], and spatial repetition effects grow with the number of successive repetitions [24]. Importantly, oculomotor capture is reduced at locations where distractors have occurred often [25,26]. Given this, our two experimental sessions differed in volatility and consequently in the distribution of run positions. Accordingly, a pooled difference between volatility regimes could arise in two ways. It could be *structural*: the same repetition-learning rule, applied to different distributions of run positions, would yield different pooled means with no change in how a given trial is processed. Or it could be *modulatory*: volatility could change processing on the current trial, in which case the regimens should differ even at the same run position. Our design permits the two alternatives to be teased apart by comparing regimens at matched run positions and by analysing distractor-absent trials, on which no distractor location can repeat and only a modulatory effect can manifest.

Three questions follow. First, does high, compared to low, volatility increase first-saccade capture even at matched run positions, as one would expect if volatility modulated overt eye-movement priority? Second, does a volatility main effect appear at a later processing stage, after the first saccade, on distractor-absent trials, where longer verification, more fixations, and fewer errors would together indicate a raised criterion for committing to a response (the *caution account*)? Third, does the trial-by-trial decline in capture as a distractor location repeats – i.e., the repetition-learning slope – itself differ across regimens, or is the same learning function applied to a different distribution of run positions? Because the same slowing of a manual response can arise from weaker evidence, from greater caution, or from both, we complement the behavioural analyses with a hierarchical drift-diffusion model [27,28] and a volatile-Kalman-filter (VKF) comparison [2]. These models ask whether the manual-response profile carries a decision-policy signature over and above a change in evidence quality.

## Methods

### Participants

We recruited 24 healthy adults (age range: 18–40 years; mean age = 26.5 years; 12 females and 12 males) for two experimental sessions (high- and low-volatility). For the volatility manipulation, the sample size was estimated based on the high- versus low-volatility effect reported by Qiu et al. [15], corresponding to a within-participant effect size of *dz* = 0.73. A two-sided paired-samples calculation with alpha = 0.05 and power = 0.80 yielded a required sample size of 17 participants. We increased the sample to 24 to account for uncertainties in eye movements. After exclusions for incomplete eye-tracking sessions (see *Awareness assessment*, below), the final analysis sample was *N* = 21; a sensitivity analysis indicates that this sample retains 80% power at α = 0.05 (two-sided) to detect within-subject paired effects of *d*z ≥ 0.64. All participants reported normal or corrected-to-normal vision. They provided written informed consent and received course credits or financial reimbursement (9 € per hour). The study was approved by the ethics committee of the Department of Psychology and Pedagogics at LMU Munich (06.04.2022).

### Apparatus

Testing took place inside a dimly lit, sound-attenuated, electrically shielded cabin. An EyeLink 1000 desktop-mounted eye tracker (SR Research, Canada) sampled the dominant eye at 1 kHz, while a 24-inch VIEWPixx/3D monitor (VPixx Technologies Inc.; 1920 × 1080 pixels, 120 Hz) rendered the search displays. A custom MATLAB R2019b script, built on Psychtoolbox and the EyeLink Toolbox [29], controlled stimulus generation, response logging, and gaze sampling and routed keyboard input to the trial loop. Throughout each session, a chin rest held participants at approximately 65 cm from the screen.

### Stimuli

Each search display arrayed 40 grey bars (0.18 × 0.81°) on a black background (RGB = 0 0 0), spaced around four concentric rings centred on the fixation cross, with radii of 1.1°, 2.2°, 3.3°, and 4.4°, respectively. Every bar carried a small notch (~0.25° tall) in either its upper or lower half, with notch position randomised and balanced within each block (Fig 1A). The bulk of these bars formed a homogeneous background of vertical (0°) elements rendered at 20% of maximum luminance. Embedded within that background, the orientation target tilted 12° to the right and carried a single upper or lower notch at the same intensity, while the orientation distractor, when present, tilted 45° to the left and carried two notches, with both stimuli matching the non-target intensity. Unlike the single-notch target, the distractor afforded neither response alternative and so could not prime response selection. Critically, target and distractor locations alike were drawn from the same set of eight positions on the second concentric ring (Fig 1A).

**Fig 1:**
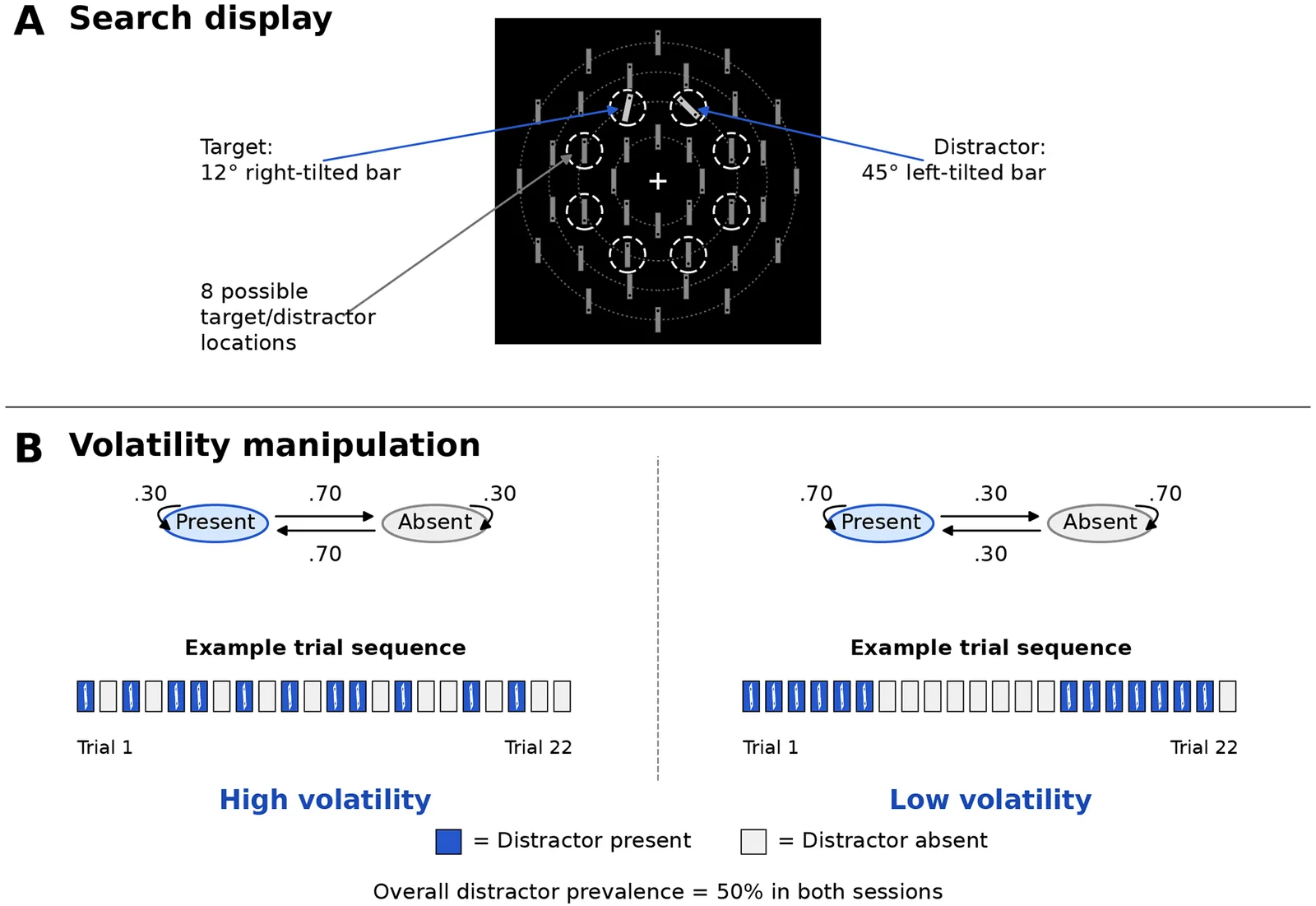
Search display and volatility manipulation. (**A)** Observers searched for a 12°-tilted target bar and reported the position of its notch (top or bottom). In some trials, a task-irrelevant 45°-tilted orientation distractor also appeared and was to be ignored. Target and distractor locations were drawn from the same set of eight equally spaced positions on the second concentric ring. (**B)** A two-state Markov process over distractor presence (Present ↔ Absent) defined the volatility regime, with transition probabilities relating trial *n* to trial *n* − 1 (PP, PA, AP, AA). In the high-volatility session (left), the state switched more often than it repeated (PP = AA = 0.30; PA = AP = 0.70); in the low-volatility session (right), it mostly repeated (PP = AA = 0.70; PA = AP = 0.30). Example 22-trial sequences below each diagram illustrate the resulting structure (filled square = distractor present; empty square = distractor absent). Overall distractor prevalence remained at 50% in both sessions, with participants completing 5–7 days apart. *Note:* PP = distractor present following present; PA = distractor absent following present; AP = distractor present following absent; AA = distractor absent following absent.

### Design

We generated each participant's trial sequence using the *Markov-chain* package in R, with a two-state Markov process over distractor presence (Present/Absent). Both sessions drew from the same generative scaffold: 1440 analysed trials, the two states equally frequent (1/2 each), overall distractor prevalence fixed at 50%, and a unique sequence drawn for every participant, which differed only in the transition matrix relating trial *n* to trial *n* − 1. The high-volatility session made switches more likely than repeats (PP = AA = 0.30; PA = AP = 0.70), producing rapid alternation; the low-volatility session reversed this structure (PP = AA = 0.70; PA = AP = 0.30), yielding longer runs of the same state (Fig 1B). Each session opened with 80 unanalyzed practice trials and continued with 9 blocks of 160 analysed trials.

In every distractor-present run, the distractor was repeated at the same location across consecutive trials, which is what makes within-session distractor-location repetition analysable. We index run position by repetition: repetition 1 denotes the first distractor-present trial of a new run (i.e., the previous trial was distractor-absent or it was the first trial of the session), and repetition 2–3 denote the second and third consecutive same-location distractor presentations within that run. Because random sequences vary in run-length distribution, we screened candidate sequences to match the count of three-trial distractor-repeat subsequences across the two volatility conditions. Three-trial repeats are the longest repetition we analysed and the level with the fewest observations, so matching them between regimes is a precondition for any volatility-by-repetition contrast. Retained high-volatility sequences contained 31–39 such subsequences, and low-volatility sequences contained 31–51 such sequences, both anchored at the same 31-subsequence floor (Fig 1B). Counts of shorter (one- and two-trial) repeats were not constrained and differed between regimes as a natural consequence of the transition matrices. Run positions beyond rep 3 (i.e., the fourth and later same-location continuations) were not pre-screened, were sparse and asymmetric across volatility regimes (high-volatility median ~12 trials/subject, min 8; low-volatility median ~72), and are excluded from the primary matched-repetition analyses. The two regimes generate intrinsically different distributions of consecutive same-location run positions, so a per-trial volatility effect can be distinguished from the run-position mixture the design imposes.

Distractor locations were not globally biased across the experiment. At the onset of each distractor-present run, the location was drawn from the eight possible target/distractor positions; subsequent trials within the run reused that location. The design therefore isolates short-term repetition-based suppression within runs rather than long-term spatial-probability learning of any high-probability location.

### Procedure

Participants performed a classic visual search task. On each trial, they searched for the target (a bar tilted 12° to the right) and indicated its notch orientation (top or bottom) by pressing a mouse button with either their right or left index finger. After responding, participants were instructed to return their gaze to the central fixation cross in preparation for the next trial.

Each trial began with a fixation cross presented for a random interval of 0.8 to 1.6 s. The visual search display remained visible until a response was issued or for up to 4 s. Participants were instructed to respond as quickly as possible without sacrificing accuracy. After an incorrect or delayed response, the fixation cross changed colour for 1 s: red for an incorrect response and blue for a slow response. Inter-trial intervals contained the fixation cross and were jittered randomly from 0.8 to 1.6 s before the next trial.

After completing both sessions, participants answered an awareness probe: *“Do you think the irrelevant left-tilted 45-degree bar changed its location more frequently from trial to trial?”*, choosing one of the following: (1) more often in Session 2 than Session 1; (2) more often in Session 1 than Session 2; (3) the location did not change obviously in either session; (4) the location frequently changed in both sessions; or (5) unsure. Eleven participants chose “unsure”, three each chose options (3) and (4), and seven chose option (1) or (2); of the latter seven, only one answered correctly.

Three participants were excluded from all analyses, two because the eye-tracking record for one of their two volatility sessions was incomplete, and one because of poor eye-tracking data quality. Exclusions were based solely on eye-tracking data quality and preceded awareness assessment. The only participant who answered correctly was among the three excluded.

### Behavioral, eye-movement, and trial-history measures

Behavioral analyses included trials with responses between 0.15 and 4 s. Error rates were computed within each participant and condition, using all valid-response trials. RT analyses used correct trials only, with an additional 1.5 × interquartile-range criterion applied to the pooled RT distribution to remove slow-response outliers; RTs are reported in milliseconds.

EyeLink EDF files were parsed in MATLAB using the Edf2Mat toolbox, which converts EyeLink events into fixation and saccade tables. Trials were segmented by fixation, search-display, and response markers. Search-period fixations were defined as EyeLink fixation events starting between display onset and the manual response. Each fixation was assigned to the nearest predefined display location, including the eight item locations and the central fixation location. The first overt orienting response was defined as the first non-central fixation after display onset. Overt oculomotor capture was operationalized as the first non-central fixation being assigned to the distractor; first-saccade-to-target was coded analogously on distractor-absent trials. First-saccade latency was measured from display onset to the launch of that first saccade. Target(-acquisition) latency was defined as the interval from display onset to the onset of the saccade that first brought the gaze to the target, indexing how quickly the target was located, before any dwell or refixation; distractor latency was defined analogously for the distractor. Additional gaze measures included the search-period fixation count and final target dwell time, defined as the duration of the final contiguous run of target fixations before response. Trial-history variables, transition type and same-location repetition position, were derived from the Markov sequence and distractor-location rules described under the Design section.

### Computational models

A hierarchical Bayesian drift-diffusion model using HSSM 0.3.0 [27] was used to assess whether the manual-response profile is better described by changes in drift rate, decision boundary, or both. The HSSM has four key parameters: drift rate *v*, boundary *a*, non-decision time *t*, and initial bias *z*. Given that the response features (top vs. bottom notch on the target) are balanced and orthogonal to the volatility manipulation, *z* was not modeled here. Four nested specifications were compared. M0 included no condition effects. In M1, drift varied with Volatility × Distractor. In M2, boundary varied with Volatility. In M3, drift varied with Volatility × Distractor, while boundary and non-decision time varied with Volatility.

This ladder isolates the two psychologically motivated decision axes, evidence quality (*v*) and response caution (*a*), that the stage account places in competition, while keeping the model space small enough to remain identifiable at the available trial counts. Boundary and non-decision time were not crossed with Distractor because both are treated as session- or task-state parameters that observers set before a trial begins and that they cannot re-set once the display reveals whether a distractor is present. Allowing *a* or *t* to vary with a within-trial factor would also compete directly with the drift term for the same RT-distribution variance and is weakly identified in practice. We therefore report M1 and M2 as interpretable one-axis projections. Full priors, posterior-predictive checks, and identifiability caveats are reported in S1 Text.

As a complementary dynamic-state analysis, we fit reduced volatility Kalman-filter [2] + DDM-style RT models that link a trial-wise latent volatility and prediction-error state to drift-like modulation, boundary-like modulation, or both. Four subjectwise architectures were compared: *Null (M0-gain-only)*, in which distractor-present gain affected drift with no latent-state modulation; *Drift-only (Mv-drift)*, in which the latent VKF state modulated drift; *Boundary-only (Ma-boundary)*, in which the latent VKF state modulated boundary; and *Full (Mva-drift-boundary)*, in which the latent VKF state modulated both drift and boundary. The observation model combined Bernoulli correctness with Gaussian RT around analytic DDM mean moments, and subject-wise fits were aggregated with AIC/BIC. Because this model is a simplified dynamic-state approximation rather than a hierarchical Wiener likelihood, we used it as a supportive architecture check on the HSSM decomposition. The full model specification is given in S2 Text, and the AIC and BIC comparison, including their divergence, is reported in the Results.

### Statistical analyses

For RT and error rate we use 2 (Volatility) × 2 (Distractor) repeated-measures ANOVAs at the trial-pooled level and 2 (Volatility) × 3 (Repetition) ANOVAs at the repetition-aware level. The same dual scheme applies to oculomotor capture, target latency, total fixations, final-target dwell, and first-saccade destination on absent trials. Distractor-absent trials contain no distractor location, so they avoid the same-location distractor-repetition confound that affects present-trial contrasts. Within-subject effect sizes are reported as *d*_*z*_, the mean of the paired differences (high-volatility minus low-volatility) divided by their standard deviation across participants; “high-minus-low” denotes this contrast throughout.

*Equivalence and Bayes-factor policy.* Any null result interpreted as evidence of absence is supplemented with both (i) a paired-samples TOST against an explicit SESOI and (ii) a default JZS Bayes factor. For the oculomotor-capture matched-repetition null we evaluated TOST at SESOI *d*_*z*_ = ±0.4 and ±0.5 and default JZS Bayes factors; because the per-repetition Bayesian/equivalence audit and the ANOVA audit used slightly different filtering details, the Results report only the bounded qualitative interpretation and not a per-repetition BF table. For the post-capture dwell null we report TOST at ±24 ms (yoked to the trial-pooled first-saccade-to-distractor effect, *d*z = 0.874) and BF_01_.

*Multiple-comparison policy.* The five-measure absent-trial Stage-3 family (RT, error rate, target latency, total fixations, final-target dwell) is the primary corrected family for the revised repetition-aware interpretation and is reported with raw, Holm–Bonferroni, and Benjamini–Hochberg FDR (*q* = .05) *p*-values, complemented by a multivariate Hotelling's *T*² on the joint 5-vector of paired differences and a Pearson correlation matrix on the across-subject Δs. Because the caution account predicts a specific direction for every measure, we complemented this omnidirectional test with a pre-specified directional contrast that asks whether the five measures move together in the predicted way. We built one *caution score* per participant as follows. For each measure, the participant's high-minus-low difference was expressed as a within-subject effect size by dividing it by the across-participant standard deviation of that difference (the *d*z scaling defined above), which places the five measures on a common, unit-variance scale. The signs were then aligned so that a positive value always denotes more caution: reaction time, target latency, total fixations, and final-target dwell entered with a positive sign, and error rate (which caution predicts should fall) entered with a negative sign. Summing the five sign-aligned values yields one caution score per participant, and a one-sample *t*-test of that score against zero (a single-degree-of-freedom test, *t* with *n* − 1 = 20 df) evaluates the one a priori prediction that the profile as a whole shifts in the caution direction. Because this contrast places all of its sensitivity on the single predicted direction, it is more powerful when the prediction holds; its signs were fixed in advance from the caution account. Other tests, including the descriptive 2 × 3 (Volatility × absent run position) ANOVAs reported in the Results, are uncorrected. They describe how the absent-trial effect distributes across run positions, without forming a separate inferential family. As a stand-alone family, the five 2 × 3 ANOVAs would not survive BH-FDR control at *q* = .05, which is one reason inference rests on the paired-*t* family and the directional composite.

For per-repetition learning slopes we fit linear mixed-effects models on subject × volatility × run-position cell means with by-subject random intercepts; an offset-only model (Volatility + Repetition) is compared against a slope-difference model (Volatility × Repetition) by likelihood-ratio test.

The hierarchical DDM and VKF analyses are reported after the repetition-aware behavioural and eye-tracking results as latent-decision descriptions of the manual-response data; the rep 1–3 truncation choice is described in the *Design* subsection.

## Results

Given that the volatility manipulation is implemented through a Markov transition probability, it can alter behavior through two non-exclusive routes. In a *structural* route, each regime imposes a different run-position mixture on the trial sequence, and an unchanged same-location repetition-learning rule, applied to a different mixture of trial histories, produces a different mean phenotype. A *modulatory* route is any residual per-trial change that remains once that mixture is equated across regimes. Accordingly, we organize the results so that each route can be evaluated separately across the search-to-response sequence. We first report the trial-pooled signatures of the manipulation, which by construction sum the two routes. We then isolate the structural route on distractor-present trials by matching same-location repetition between regimes, asking whether the pooled capture effect survives once the run-position mixture is equated across regimes and whether volatility alters the local learning slope. Next, we turn to the modulatory route on distractor-absent trials, which involve no distractor-location repetition and so expose any residual per-trial effect directly, in later target acquisition, verification, and response. Finally, we fit hierarchical-Bayesian and reduced VKF latent-decision models to manual responses as descriptive decompositions of later choice-related structure.

### The pooled phenotype: Structural and modulatory routes combined

#### Trial-pooled RT, error rates, and oculomotor capture.

A 2 (Distractor condition: absent vs. present) × 2 (Volatility: high vs. low) repeated-measures ANOVA on correct-trial RTs showed the expected distractor-interference effect: responses were slower when the singleton distractor was present than when it was absent (1129.7 vs. 976.0 ms), *F*(1, 20) = 178.07, *p* < 0.001, η_p_² = 0.90. The main effect of volatility was marginal, with slower responses in high- vs. low-volatility sessions (1107.3 vs. 998.5 ms), *F*(1, 20) = 4.15, *p* = 0.055, η_p_² = 0.17. The Distractor × Volatility interaction was not reliable, *F*(1, 20) = 0.68, *p* = 0.420, η_p_² = 0.03 (Fig 2A). Errors were rare overall (≈ 2.2%) and showed a volatility main effect, *F*(1, 20) = 6.78, *p* = 0.017, η_p_² = 0.25 (1.7% high vs. 2.7% low, Fig 2B), with neither the distractor main effect nor the interaction reliable (*p*s > 0.397).

**Fig 2:**
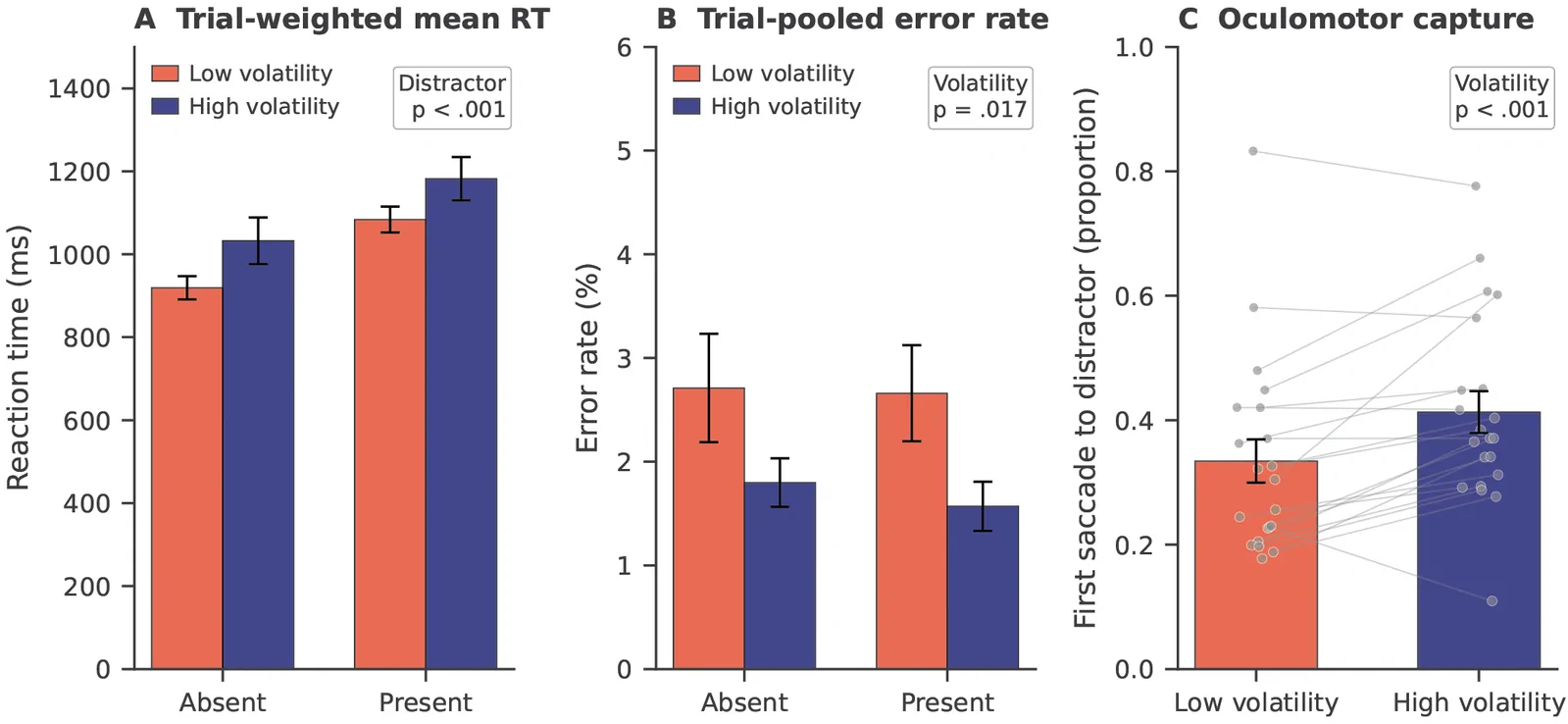
Trial-pooled signatures of the volatility manipulation. **(A)** Mean RT in the four cells of the Volatility × Distractor design; the in-panel label marks the significant Distractor main effect (*p* < 0.001), while the Volatility main effect was marginal (*p* = .055) and the Distractor × Volatility interaction was not significant (*p* = .42). **(B)** Error rate in the same four cells; the in-panel label marks the Volatility main effect (*p* = .017), with neither the Distractor main effect nor the interaction reliable. **(C)** Proportion of first saccades to the distractor on distractor-present trials, trial-pooled: higher with high vs. low volatility (*p* < .001). These pooled summaries motivate the repetition-aware decomposition reported below.

Pooling across same-location run positions, the proportion of first saccades to the distractor on present trials was higher in high- vs. low-volatility sessions (41.3% vs. 33.4%; + 7.9 percentage points, pp), paired *t*(20) = 3.94, *p* < 0.001, *dz* = 0.86 (Fig 2C). In a conventional analysis, this pooled difference would be interpreted as a per-trial volatility effect on initial overt oculomotor allocation. The repetition-aware analysis below tests that interpretation.

The pooled manual and oculomotor results diverge (Fig 2). High-volatility slowed responding and reduced errors, yet it did not reliably modulate distractor interference (RT_present_ - RT_absent_) in the omnibus 2 × 2 ANOVA above, whereas first-saccade-to-distractor was clearly higher under high than under low volatility (Fig 2C).

### The structural route: Present-trial capture reduces to the repetition mixture

The trial-pooled present-trial capture contrast sums the two routes by construction. Each pooled mean is a regime-specific mixture of the same-location run positions. Same-location run position varies by an order of magnitude across present-trial means under the two volatility regimes, and several distractor-suppression measures depend strongly on run position. The structural route alone could therefore in principle produce the pooled present-trial capture difference. We therefore re-examined the present-trial capture contrast with run position matched between regimes to test whether any residual per-trial effect remained in initial overt oculomotor allocation and whether volatility altered the local repetition-learning slope.

#### The repetition mixture imposed by the Markov design.

The two volatility regimes generate different distributions of consecutive same-location run positions on present trials. In high-volatility sessions, the distractor repetition pool is approximately 70% rep 1 / 21% rep 2 / 9% rep 3 + ; in low-volatility sessions, the split is 31% / 23% / 47%. Oculomotor capture is intrinsically higher at rep 1 than at rep 3 (combined-volatility means ≈ 47% vs. ≈ 26%), so any pooled present-trial mean compares a repetition-1-heavy mean under high volatility with a repetition-3-heavy mean under low volatility. The + 7.9-pp pooled capture difference is consistent with this repetition mixture alone, without any per-trial volatility effect on capture. The next analyses separate the structural mixture from any per-run-position volatility effect.

#### Present-trial effects at matched same-location repetition.

A 2 (Volatility) × 3 (Repetition: rep 1, rep 2, rep 3+) repeated-measures ANOVA on the proportion of first saccades to the distractor returned no reliable volatility main effect, *F*(1, 20) = 0.28, *p* = .604, η_p_² = .014; a strong monotonic repetition decay, *F*(2, 40) = 84.04, *p* < .001, η_p_² = .81; and a non-reliable interaction, *F*(2, 40) = 1.31, *p* = .282 (Fig 3A).

**Fig 3:**
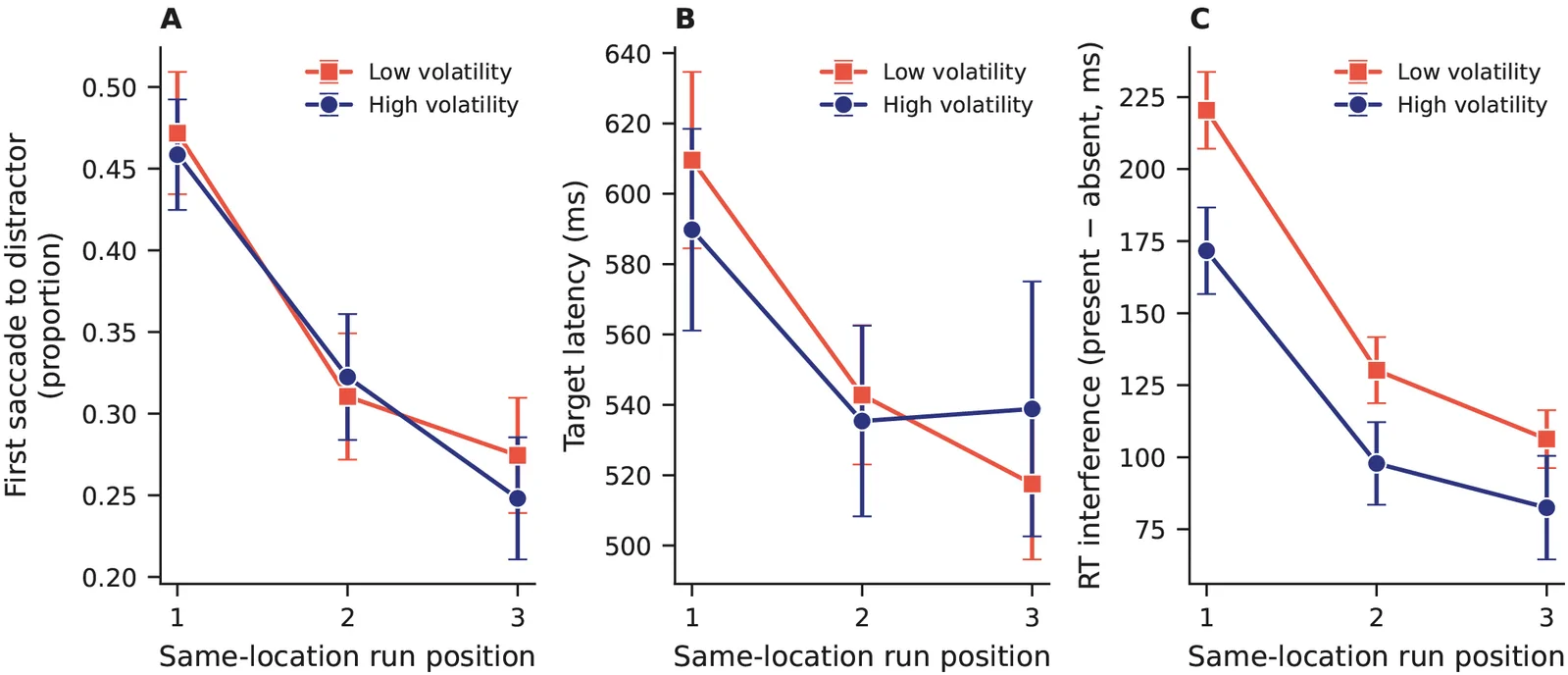
Repetition-driven learning across volatility regimes. Same-location run position on the *x*-axis; high-volatility (blue) and low-volatility (orange) sessions overlaid. **(A)** Proportion of first saccades to the distractor. **(B)** Target latency (ms), the time to first direct gaze on the target. **(C)** RT-defined distractor interference (ms). Repetition produced a strong monotonic decay on every measure, with no detectable volatility-related slope difference (LMM slope-difference LRT *p* = .692, .256, .174 for A, B, **C)**.

Bayesian and frequentist equivalence tests gave a bounded null interpretation. Bayes factors favoured the null overall (rep-equal pooled BF_01_ = 3.88), with moderate support at rep 1 (BF_01_ = 3.98), rep 2 (BF_01_ = 3.50), and rep 3+ (BF_01_ = 2.14); the corresponding TOST at SESOI *d*z = ±0.4 was inconclusive in every cell, reflecting the limited sensitivity of *n* = 21. Post-capture engagement (i.e., dwell on the distractor once it was fixed) likewise did not differ across regimes (S3 Text).

The analogous 2 × 3 ANOVA on present-trial RT yielded no reliable volatility main effect, *F*(1, 20) = 2.10, *p* = .163, η_p_² = 0.095, although per-repetition gaps were directionally consistent with a small slowing under high volatility (+65 / + 81 / + 90 ms across reps 1/2/3 + ; all *p* > 0.13). When RT-defined distractor interference was considered under repetition-equal weighting (the mean across reps 1, 2, and 3 + instead of across raw trials), the cost was smaller under high vs. low volatility, *F*(1, 20) = 10.22, *p* = 0.005. This repetition-equal cost reflects the contrast between matched present-trial run positions and the absent-trial baseline, decomposed further as inter-trial appearance and lingering switch costs in S5 Text. The trial-pooled present-trial signatures reported above are therefore consistent with the repetition-mixture imposed by the Markov manipulation, and matching run position leaves no detectable per-trial effect on capture.

#### Rate-invariant repetition learning.

Linear mixed-effects models on subject × volatility × run-position cell means tested whether volatility shifts the per-repetition learning slope. On every measure, the slope-difference term was non-significant. First-saccade-to-distractor: β_int_ = −0.007, χ²(1) = 0.16, *p* = .692. Target latency: β_int_ = +20.5 ms/rep, χ²(1) = 1.29, *p* = .256. RT-defined distractor interference: β_int_ = +12.5 ms/rep, χ²(1) = 1.84, *p* = .174. Each measure showed a strong repetition main effect (e.g., first-saccade-to-distractor 0.46 → 0.27 across reps 1/2/3 + ; RT-defined cost 196 → 94 ms). We therefore found no detectable volatility-related slope difference in the local learning rule that converts same-location repetition into spatial suppression (Fig 3).

### The modulatory route: Distractor-absent verification and caution

Matching same-location repetition left no detectable per-trial capture effect on present trials. Distractor-absent trials test for a modulatory effect directly: with no distractor in the display, no location can repeat, and any regime difference is a per-trial effect unconfounded by repetition.

#### The distractor-absent profile.

Four of the five measures shifted in a converging direction under high volatility (Fig 4A; per-participant data in S6 Text and S3 Fig). RT was longer (+113.3 ms), *t*(20) = 2.08, *p* = 0.051, *d*z = +0.45. Errors were rarer (−0.9 pp), *t*(20) = −2.46, *p* = 0.023, *d*z = −0.54. Total fixations rose by +0.34, *t*(20) = 2.75, *p* = 0.012, *d*z = +0.60. Final target dwell also rose by +80.5 ms, *t*(20) = 2.05, *p* = 0.054, *d*z = +0.45. By contrast, target latency did not differ across regimes (+6.9 ms, *t*(20) = 0.41, *p* = 0.684, *d*z = +0.09), indicating the time to first direct gaze on the target was unchanged. Since neither the destination nor the latency of the first saccade differed between regimens (S7 Text), the profile must arise after the first saccade.

**Fig 4:**
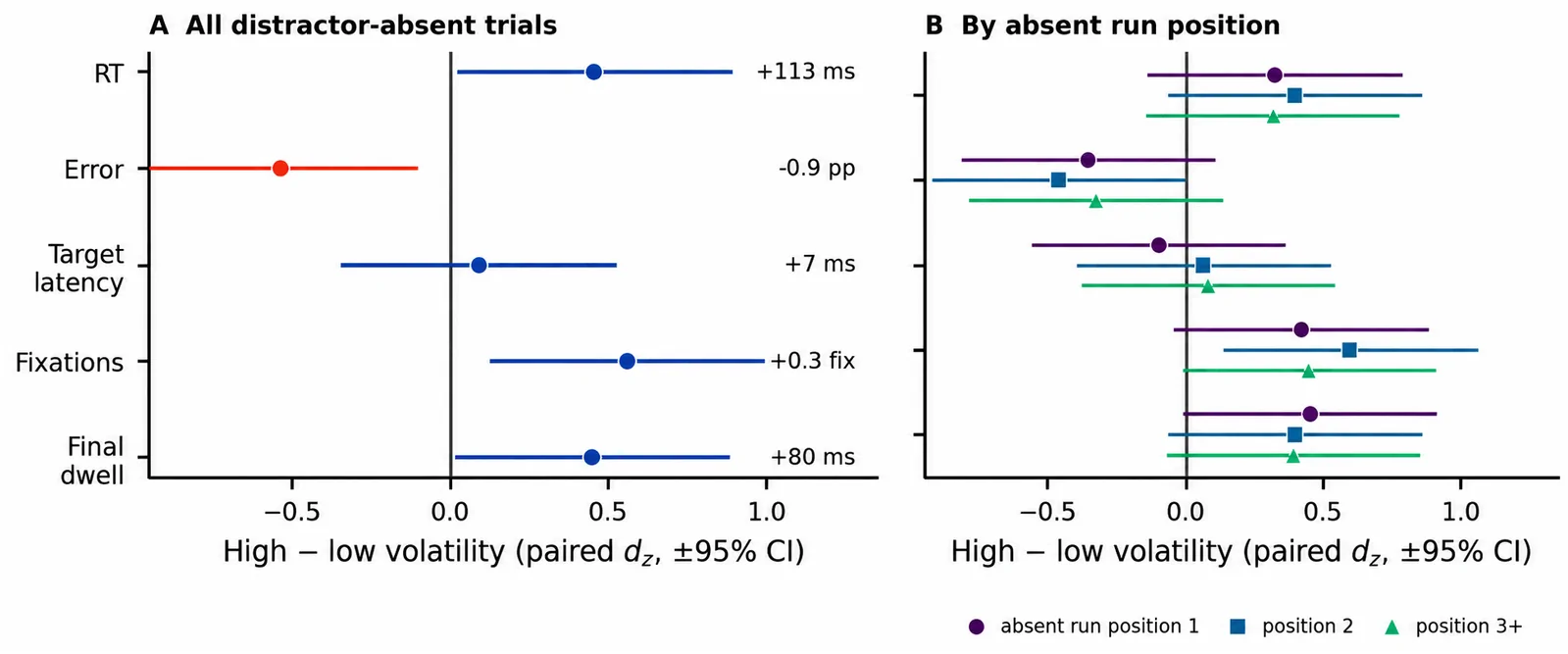
Distractor-absent volatility profile. **(A)** Paired high-minus-low volatility contrasts for the five distractor-absent measures (reaction time, error rate, target latency, total fixations, and final-target dwell) in within-subject dz units (points, ± 95% CI); right-hand labels give the corresponding raw high-minus-low differences in native units. Error rate, total fixations, final-target dwell, and RT shift in a converging direction. No measure survives family-wise correction. **(B)** The same contrasts computed separately at absent run positions 1 (the first absent trial after a distractor-present trial), 2, and 3 + . A task set carried over from the preceding present trial predicts the largest gap at position 1, with decay thereafter; however, the observed profile is flat across positions (S8 Text and S2 Table). Per-participant paired summaries for each measure are shown in S6 Text (S3 Fig).

#### A directional test of the joint shift.

The caution account predicts that every measure shifts toward more cautious verification. A pre-specified directional composite of the five sign-aligned measures, each scaled to *d*z units (see Methods), was reliable, *t*(20) = 3.01, *p* = .007. The version restricted to the three measures drawn from the eye-movement record (target latency, total fixations, and final-target dwell) was also reliable, *t*(20) = 2.31, *p* = .032. The directional effect therefore does not depend on the response-time signal to which the decision model is also fitted. The omnidirectional alternative, Hotelling's T², was non-significant, T² = 13.95, *F*(5, 16) = 2.23, *p* = .101, and no single measure remained significant after Holm–Bonferroni or Benjamini–Hochberg FDR correction at α = .05. The omnibus test spreads power over all five dimensions, whereas the measures are correlated and co-directional (S6 Text). The joint shift in the predicted direction is therefore reliable, although no individual measure remains significant after family-wise correction.

#### The profile across the absent run.

High volatility produces more present-to-absent transitions, so the profile could reflect a task set carried over from the preceding distractor-present trial. Such a task set decays with distance from the transition, which predicts the largest high-minus-low gap at *absent run position* 1, the first absent trial after a transition. A sustained adjustment to the switch probability predicts a flat gap, because under a first-order Markov process that probability is the same on every absent trial.

The gap was flat (Fig 4B; S8 Text and S2 Table). No measure showed a Volatility × Run-position interaction (all *F*(2, 40) ≤ 1.71, all *p* ≥ .193, all η_p_² ≤ .08). The decay contrast, the gap at run position 1 minus the gap at run position 3 + , was close to zero for RT, error rate, total fixations, and final-target dwell, with 95% confidence intervals that span zero. For target latency, the decay contrast was negative (−14.9 ms [−29.0, −0.7]), indicating that the gap grew across the run. These tests bound a decaying component, without excluding it. With *n* = 21, the interaction test has 80% power only for effects of η_p_² ≥ .21, whereas the observed interactions ranged from η_p_² = .01 to .08. They do show that the profile is not concentrated on the post-transition trial, the signature that would distinguish a decaying task set from a sustained adjustment to state uncertainty.

### Latent-decision counterparts in manual responses

#### Hierarchical DDM provides a descriptive drift/boundary decomposition.

The manual RT/error profile was also fitted with a hierarchical Bayesian drift-diffusion model (HDDM-style DDM, implemented in HSSM). Model comparison favoured the full model M3, in which drift rate varied with Volatility × Distractor and both decision boundary and non-decision time varied with Volatility (Fig 5C). M3 outperformed the drift-only, boundary-only, and null models (stacking weight = 0.71; Δelpd_loo_ relative to M3 = 383 for M1, 686 for M2, and 953 for M0). This indicates that the manual-response likelihood contains information about both drift-related and boundary-related volatility effects.

**Fig 5:**
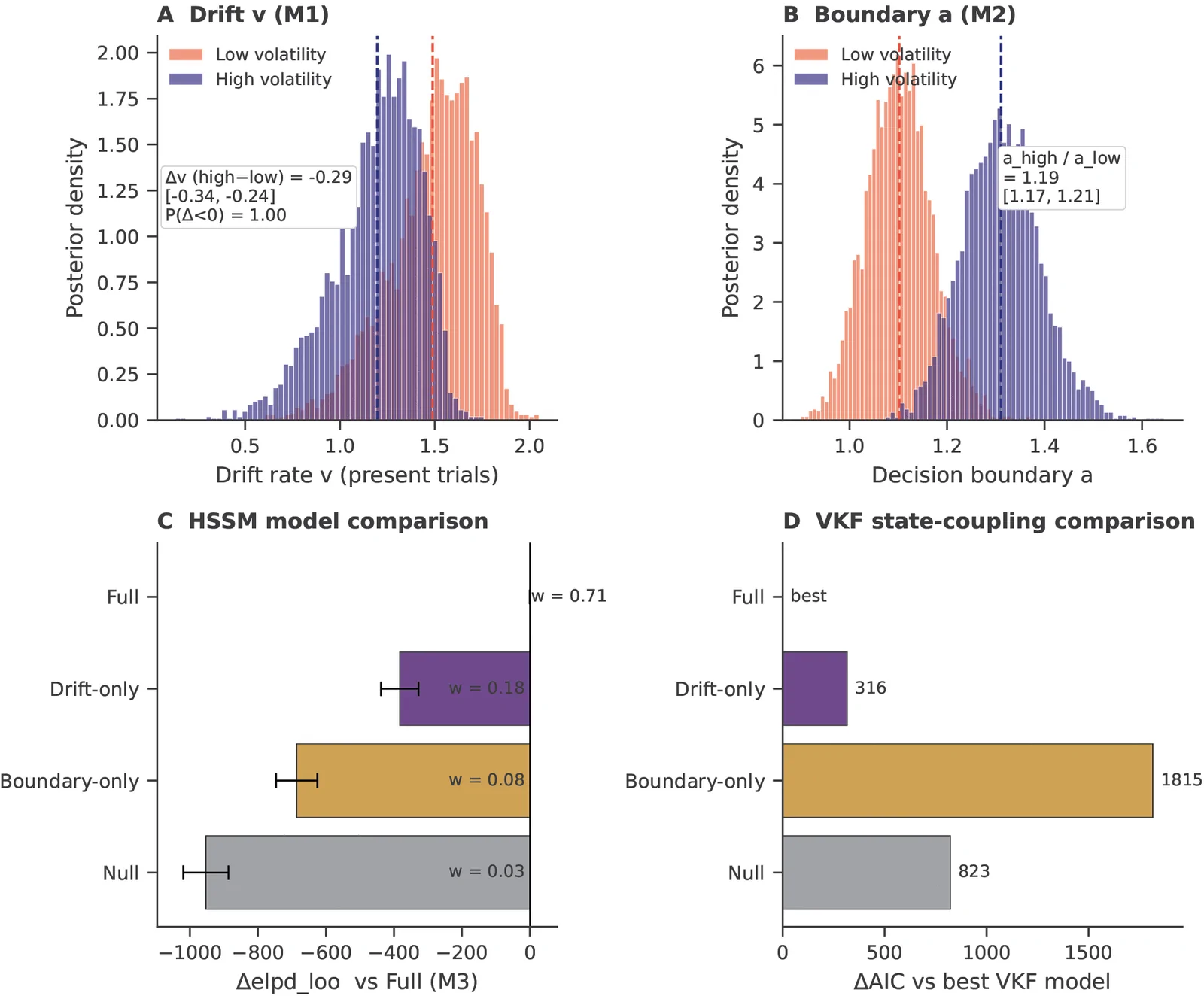
Computational modelling of manual responses. **(A)** Posterior distributions for present-trial drift rate *v* in the HSSM drift-only model M1. **(B)** Posterior distributions for decision boundary *a* in the HSSM boundary-only model M2. **(C)** HSSM PSIS-LOO model comparison, plotted as Δelpd_loo relative to the full model (M3); more negative values indicate poorer expected predictive accuracy than the full model. **(D)** Reduced VKF + DDM-style model comparison, plotted as ΔAIC relative to the best VKF model. Panels C and D use the same row order (top→bottom: Full, Drift-only, Boundary-only, Null) and the same colour scheme per architecture, so the two model families can be read against each other directly. The HSSM provides the primary latent-decision decomposition; the VKF panel provides a supportive check on the dynamic-state architecture.

Because the full model's cell-level parameter magnitudes lie on a known parameter-identifiability ridge (drift rate v, boundary a, non-decision time t), we use the lower-dimensional models as interpretable projections of the two decision axes. The full model does not provide a uniquely identified joint decomposition. In the drift-only projection (M1), drift was lower under high than under low volatility on both trial types, Δv_high−low_ = −0.29, 95% HDI [−0.34, −0.24] on distractor-present trials (Fig 5A), and −0.52 [−0.59, −0.46] on distractor-absent trials. The distractor-related drift cost (present minus absent) was accordingly smaller under high volatility (−0.46 [−0.51, −0.41]) than under low volatility (−0.69 [−0.75, −0.63]).

In the boundary-only projection (M2), the decision boundary was higher under high volatility, a_high_ / a_low_ = 1.19, 95% HDI [1.17, 1.21] (Fig 5B). The boundary component provides a latent-decision counterpart to the distractor-absent verification/caution profile. The drift component shows that the manual RT distribution reflects more than a boundary shift. The drift reduction is also present and larger on distractor-absent trials, which carry no repetition mixture. Thus, it is a per-trial effect of volatility on evidence accumulation in the manual decision and cannot be attributed to trial history. Because the distractor-related drift cost was smaller under high volatility, it also does not indicate an impairment of early distractor filtering.

#### Reduced VKF dynamic-state comparison.

A reduced VKF + DDM-style RT comparison asked a related dynamic-state question: does a trialwise latent volatility/prediction-error state improve prediction through drift-like modulation, boundary-like modulation, or both? The four VKF models are named in parallel to the HSSM specifications M0-M3 (Null, Drift-only, Boundary-only, Full; full equations in Section S2.2 in S2 Text). The full drift/boundary model provided the best aggregate AIC across 60,255 RT-bounded trials from 21 subjects (AIC = 80245.84), followed by the drift-state model (ΔAIC = 316.26), the gain-only baseline (ΔAIC = 822.57), and the boundary-state model (ΔAIC = 1815.18; Fig 5D). Subject-wise diagnostics showed that the full model improved negative log likelihood relative to the gain-only baseline for 21/21 subjects (median ΔNLL = −5.93), while the drift-state and boundary-state models improved fit for 19/21 and 16/21 subjects, respectively.

The VKF comparison corroborates the HSSM at the architectural level. Its role is limited to that level because it uses a simplified Bernoulli-correctness plus Gaussian-RT likelihood with subjectwise maximum-likelihood fits, whereas the HSSM uses a hierarchical Wiener likelihood. On the question of model architecture, the two model families converged. AIC indicated that the manual RT data were best described when both drift-like and boundary-like modulation were allowed. BIC instead favoured the Null baseline (BIC = 82014.08 for Null vs. 82326.31 for Full), consistent with the stronger penalty for the additional subject-wise parameters at this trial count. The two model families converge on the conclusion that the manual-response data involve both drift and boundary modulation. The VKF adds architectural support to that conclusion without localising boundary setting or early gating.

## Discussion

Environmental volatility increased response caution. The effect appeared late in the search-to-response sequence, on target verification and on the criterion for committing to a response, well after the first overt shift of gaze. The findings answer the three questions raised in the Introduction as follows. First, once we matched consecutive same-location run positions across regimens, first-saccade-to-distractor no longer differed. The + 7.9-pp rise in the pooled contrast is fully accounted for by the run-position mixture imposed by the manipulation, with no residual effect on overt capture. Second, on distractor-absent trials, where distractor-location repetition was absent, high volatility produced longer verification and dwell, more fixations, and fewer errors, while target acquisition remained unchanged. The manual response carried both decision boundary- and drift-related structure. Third, the same-location repetition-learning slope held constant across regimens. The manipulation thus affected behavior by two routes. In the *structural* route, an unchanged learning rule is applied to a different distribution of run positions and so produces different mean behavioral patterns; in the *modulatory* route, volatility changes processing on the current trial, the residual effect once that mixture is held fixed. In this design, the structural route accounts for the pooled volatility effect, while the modulatory signature emerges downstream during verification and response, where the distractor-absent measures show convergent changes.

The first saccade plays a specific role in this account. Its destination is itself a decision, the earliest overt resolution of a competition among salience, task goals, learned priority, and saccade planning, and its latency marks when that decision was enacted. Everything after it, from target acquisition and corrective orienting to verification and response preparation, belongs to later stages. Therefore, the first-saccade measures index overt oculomotor capture only. High volatility drew more first saccades to the distractor in the pooled data, but the difference vanished once run position was matched, with first-saccade latency showing the same pattern (S4 Text and S2 Fig). The first (*overt*) saccade cannot adjudicate *covert* selection, peripheral sensory gain, or the full machinery of saccadic choice, so those questions remain open [18–20]. These first-saccade findings do not require saccadic and manual decisions to be separate systems, although the dissociation we observe aligns with evidence that perceptual and action-oriented spatial read-outs can dissociate [30].

The later profile fits a precision-weighting account of volatility. Adaptive frameworks hold that volatile environments lower confidence in long-run state estimates and raise the weight on recent evidence [1–3,6,31]. In sequential-sampling models, greater response caution is expressed as a higher decision boundary, requiring more accumulated evidence before commitment [32]. The distractor-absent data show this pattern. Final-target dwell lengthened, fixations increased, and errors fell. No single measure remains significant after family-wise correction, and target acquisition was unchanged, but the measures shift together in the predicted direction reliably enough to reject a null of no joint effect. The boundary-only HSSM projection (*a*_high_/*a*_low_ = 1.19) gives that beh*a*vior*a*l profile a latent-decision counterpart, and the full model shows that the manual response also carries drift-related structure. The account is falsifiable, with the prediction that manipulating caution directly through speed-accuracy instructions or accuracy-weighted payoffs should affect the distractor-absent verification measures more strongly than matched-repetition capture or the repetition-learning slope.

Effects of volatility have been reported across several cognitive domains, and the present findings are consistent with this broader pattern. Volatile contexts raise learning rates in reinforcement learning [1,2], reshape how uncertainty is tracked and weighted in anxiety and mood [3], prompt precision adjustments across levels of the perceptual inferential hierarchy [4,6,31,33,34], and shift commitment thresholds in sequential-sampling models of choice [28,32]. In the present visual-search task, with overall distractor frequency held constant, the volatility-related adjustment emerged downstream of overt capture, during later verification and response commitment.

The latent-decision models decompose the manual RT and error distributions into an evidence-quality component (drift rate) and a response-criterion component (boundary separation), which raw RT cannot separate. Adding boundary and non-decision-time structure to a drift-only model produced a large gain in predictive accuracy, and the boundary was 19% higher under high volatility, matching the slower but more accurate response. The full model also outperformed a boundary-only model, and drift rate was lower under high volatility on both trial types. The absent-trial estimate is informative because absent trials carry no repetition mixture: the drift reduction is therefore a per-trial effect of volatility on evidence accumulation in the manual decision and cannot be attributed to trial history. Its pattern also argues against a capture-side origin, because the distractor-related cost in drift was smaller under high volatility than under low volatility, the opposite of what an impairment of early distractor filtering would produce. Therefore, the manual response carries two per-trial adjustments: a raised criterion and a reduced rate of evidence accumulation. Both are consistent with an observer who trusts the current state less and requires more evidence before committing. A diffusion model cannot assign the drift component to a single perceptual stage. The complementary VKF comparison supports the same architecture without settling the parameter question. AIC favoured the fuller drift-and-boundary architecture and BIC the learner baseline; because the VKF likelihood is a simplified stand-in for the hierarchical Wiener model used in HSSM, we treat it as corroboration of the architecture only. The two models converge on the conclusion that the manual response carries a decision-policy contribution alongside a change in evidence quality.

Repetition learning ran at the same rate in both regimens, on every measure we examined. The learning itself has precedents at two-time scales. Across consecutive trials, priming of pop-out leaves feature- and position-specific traces that accumulate over repetitions [35,36]. Interference is reduced when a distractor repeats at its previous location [8], and repeating a distractor feature reduces distractor-evoked capture [24] and speeds response [37], though one-back location repetition does not benefit performance in every design [38]. Over longer timescales, experience with a salient distractor lowers the priority of its likely location or of its defining feature dimension [39–42]. Neither line of literature makes such local updating conditional on the higher-order structure of the trial sequence, and our data showed no such dependency. Learning proceeded at the same rate under a volatility manipulation that observers could barely report. Volatility therefore acted through the structural route by changing how often each run position occurred without changing how each run position was processed.

Our repetition effect is related to serial dependence but differs in what is measured. Serial dependence is typically studied as a bias in perceptual experience or continuous report [43–46], whereas our eye-movement, response-time, and error measures index performance rather than how the display was perceived. The point of contact is decisional because recent choices can carry over to current choices even when perceptual and post-perceptual history effects dissociate [47–49], and the repetition in our task could operate through the same decisional channel onto oculomotor selection or response policy. Our design cannot separate such carryover from spatial suppression and statistical learning, so serial dependence remains an adjacent account to be tested.

One alternative account must be considered. Because high volatility produces more present-to-absent transitions, the distractor-absent profile might reflect a task set carried over from the preceding distractor-present trial, rather than an adjustment to volatility itself. This account has two versions. In the first, the carried-over task set decays with each successive absent trial. This version predicts a gap concentrated on the first absent trial after a transition. The run-position analysis (Fig 4B; S8 Text and S2 Table) shows no such concentration: the decay contrasts are close to zero, with confidence intervals that bound the decay the data allow. In the second, observers are more cautious on absent trials under high volatility because they assign a higher probability that a distractor is about to reappear. This version is a description of how a volatility effect operates. Overall distractor prevalence was 50% in both regimes, so the only quantity that raises the expectation of a distractor on an absent trial is the probability that the state switches back, which is the transition probability that defines volatility. Because that probability is constant across absent run positions under a Markov design, this account predicts exactly the flat, elevated profile we observed. The run-position tests are not powered to exclude a small decaying component. They do exclude the specific temporal signature that would distinguish a decaying task set from a sustained adjustment to state uncertainty. Because the absence of a distractor is harder to verify than its presence, the adjustment is expressed where verification is most demanding, on distractor-absent trials. The effect is therefore best described as post-selective verification under asymmetric state uncertainty. A symmetric design, in which two equally verifiable task states alternate under different volatilities, would permit testing whether the same criterion shift appears without that asymmetry, as a global caution parameter would predict.

These results extend recent volatility work in distractor-bias designs. Qiu et al. [15] varied volatility over a strong long-term spatial bias and found it modulated how firmly observers deployed that learned prior. We removed the long-term prior, so that suppression had to be built from short-term repetition. Here the structural route appears as a run-position mixture effect, whereas in Qiu et al. it appeared as modulation of a spatial prior. Together the two studies suggest that volatility acts on the learned regularity that is locally available, and that in the present design the clearest residual signature lies later in the search-to-response sequence. The finding also complements Bogaerts et al. (2022) and van Moorselaar and Slagter [13,14], who showed that distractor regularities at different timescales modulate suppression. By contrast, we isolate volatility itself with overall distractor frequency fixed. It also aligns with recent calls to distinguish initial selection, post-capture engagement, target verification, and response commitment [23], as our measures map onto successive stages and volatility-related effects emerge later in the sequence.

Several extensions would strengthen the dissociation. The sample of 21 observers provided sensitivity primarily to medium-sized effects. A larger sample would increase the precision of the matched-repetition capture estimate, the multivariate distractor-absent profile, and the equivalence test at small-effect bounds. The HSSM and VKF fits recover useful latent-decision axes but do not uniquely partition the full *v–a–t* space. We therefore rely on their convergence with the eye-tracking analyses and treat the parameter magnitudes as descriptive. Volatility here governed the binary presence or absence of the distractor. Defining it over distractor location or feature would provide a more stringent test, particularly because between-dimension filtering can follow a partly distinct logic [11]. Awareness was poor, with none of the 21 participants included in the analysis correctly identifying the higher-volatility session. The observed effects therefore did not depend on explicitly reportable knowledge of the volatility manipulation.

A design principle follows from the structural route. Any volatility manipulation that operates through the transition probability of a binary latent state produces different run-position distributions across the two regimes. Whenever the dependent measure tracks run position, and nearly every short-term distractor-suppression measure does [22,35,39], the pooled volatility contrast will confound the per-trial effect with the repetition mixture that the design itself creates. Pooled contrasts still describe overall behavioral differences between volatility conditions, but mechanistic inference about per-trial effects requires conditioning on run position. The same caution applies wherever state-persistence manipulations generate state-dependent trial dynamics, from reward paradigms that vary contingency volatility to cued-task-switching designs that vary switch probability.

In sum, the study provides a stage-sensitive account in which environmental volatility increased response caution and left oculomotor capture without detectable change at matched repetition. What appears to be an effect on attentional capture partly reflects the repetition mixture imposed by the manipulation, whereas the detectable per-trial effect emerges later during target verification and response commitment. It reflects a decision-policy adjustment downstream of capture. First-saccade capture indexes overt orienting and does not measure sensory gain, so the matched-repetition null constrains an early-capture account without excluding covert or sensory effects beyond the present measurements. Faced with a more volatile environment, observers made more fixations, dwelt longer on the target before responding, responded more slowly, and made fewer errors. This is the signature of adaptive caution in visual search induced by volatility.

## Supporting information

S1 Text Hierarchical Bayesian drift-diffusion model. (DOCX)

S2 Text Reduced VKF dynamic-state comparison. (DOCX)

S3 Text Post-capture dwell duration. (DOCX)

S4 Text Present-trial first-saccade latency by same-location repetition. (DOCX)

S5 Text Inter-trial switch costs in manual RT. (DOCX)

S6 Text Distractor-absent profiles: per-participant data and dependence among measures. (DOCX)

S7 Text Distractor-absent first-saccade allocation. (DOCX)

S8 Text Task-set inertia: distractor-absent profile across absent-run positions. (DOCX)

S1 Fig Posterior-predictive RT distributions from the three hierarchical DDMs — M3 (full; red, solid), M1 (drift-only; orange, dashed), and M2 (boundary-only; purple, dotted) — overlaid on observed RTs (blue), separately for the four Volatility × Distractor cells. For each model, predictive datasets were forward-simulated from eight joint posterior draws (fixed effects and by-subject offsets taken from the same draw; the two absorbing bounds separated by 2*a* — HSSM’s ±*a* parameterisation — with starting point *z*· 2*a*; σ = 1, dt = 1 ms), matched to the empirical design and pre-processing (RT ∈ [0.15, 4] s, correct trials). M3 reproduces the location, spread, and right tail of the observed distributions in every cell (predicted cell means within 9–23 ms of the empirical means) despite sitting at an extreme point of the *v*–*a*–*t* ridge — the identifiability argument of S1.3 in practical form. The marginal projections behave as their structure dictates: M1, which carries the cell structure through drift alone, tracks the cell means nearly as well, whereas M2, in which neither drift nor boundary varies with Distractor, predicts near-identical present and absent distributions within each regime and so misses the interference structure (S1.4). Cell magnitudes in the main analyses are therefore quoted from M1 and M2 as one-axis projections.
(TIF)

S2 Fig Present-trial first-saccade measures as a function of same-location run position, plotted in the format of Fig 3. (A) First-saccade latency across all first saccades. (B) First-saccade latency restricted to saccades landing on the distractor. (C) Proportion of first saccades landing on the distractor (oculomotor capture), reproduced from Fig 3A for direct comparison. Points are condition means; error bars are ± 1 within-subject SEM. High-volatility sessions in blue, low-volatility in orange.
(TIF)

S3 Fig Per-participant paired high/low volatility summaries for the five distractor-absent measures summarised in Fig 4: (A) reaction time, (B) error rate, (C) target latency, (D) total fixations, and (E) final-target dwell. Bars show condition means; grey lines link the same participant across volatility sessions; error bars are ± 1 SEM. Subjects whose values fall outside the plotted range are drawn as small triangles at the corresponding axis edge (▲ above, ▼ below). Every participant shown contributed to every statistical test reported; no data point was removed on the basis of its plotted value. Brackets give the uncorrected paired high-versus-low contrast within each panel (* *p* < .05; n.s. = not reliable); no measure survives family-wise correction.
(TIF)

S1 Table Subject-conditional posterior-predictive check of the marginal models M1 and M2 (*n* = 20 simulated full-design datasets per model). *Empirical* is the observed trial-pooled (high − low) difference in RT-defined distractor interference (RTpresent − RTabsent); it is identical for both rows because it is a property of the data, not the model. Predictive medians and intervals are quantiles across the 20 simulated datasets.
(DOCX)

S2 Table Distractor-absent volatility gap (high − low) at absent run positions 1, 2, and 3 + ; the Volatility main effect and the Volatility × run-position interaction from 2 × 3 repeated-measures ANOVAs; and the decay contrast, the gap at position 1 minus the gap at position 3 + , in native units and in *d*z units (paired *t*-test; *n* = 21). A carried-over task set predicts a positive decay contrast for RT, target latency, total fixations, and final-target dwell, and a negative one for error rate.
(DOCX)
