# Supplementary material for: Environmental volatility is associated with adaptive caution in visual search
Source: PLoS Comput Biol. 2026 Oct 21;22(10):e1014310. doi: 10.1371/journal.pcbi.1014310 (PMC13712733; doi:10.1371/journal.pcbi.1014310)
Supplement: Supplementary file 1 [file pcbi.1014310.s001.docx]

## S1 Text. Hierarchical Bayesian drift-diffusion model

### S1.1 Model family and notation

The drift-diffusion model (DDM; Ratcliff & McKoon (2008)) treats each correct/incorrect choice as the outcome of a noisy accumulator that drifts at rate *v* between two absorbing boundaries at ±*a*, following the HSSM/ssm-simulators parameterization in which a is half the classic (HDDM-convention) boundary separation. The accumulation starts from a relative point *z* ∈ (0, 1), with non-decision time *t* added to encoding/motor latencies. We fit an HDDM-style hierarchical DDM using HSSM 0.3.0 (Fengler et al., 2022) with the analytical Wiener first-passage-time likelihood:

| **Model** | **Parameters that vary** | **Design contrast(s)** | **Fixed-effect df** |
| --- | --- | --- | --- |
| M0 (null) | none | intercept-only on *v*, *a*, *t* | 0 |
| M1 (drift-only) | *v* | Volatility × Distractor (2 × 2) | 3 |
| M2 (boundary-only) | *a* | Volatility (2 levels) | 1 |
| M3 (full) | *v*, *a*, *t* | *v* ~ Volatility × Distractor; *a* ~ Volatility; *t* ~ Volatility | 5 |

Volatility ∈ {high, low} and Distractor ∈ {present, absent}. Reference levels are volatility = high and distractor = absent, so that volatility coefficients on *v*, *a*, and *t* encode the (low − high) contrast and the *v* distractor coefficient encodes the (present − absent) contrast. All four models include by-subject random intercepts on *v*, *a*, and *t*. The relative starting point z and the lapse mixture are shared across the four specifications. All four models were fit to the same random subsample of 200 trials per subject × Volatility × Distractor cell (16,800 trials in total; fixed subsampling seed), which keeps the four hierarchical JAX/NumPyro fits tractable on the available hardware. Per-cell raw counts are ~670–760, so the subsample is a uniform random draw within every cell. Posteriors were sampled with NUTS via NumPyro/JAX (4 chains, 1000 tuning + 1000 sampling iterations per chain, target acceptance 0.95). Convergence was assessed via R-hat (≤ 1.01 required) and effective sample size (≥ 400 bulk and tail required). Model comparison used leave-one-out cross-validation with Pareto-smoothed importance sampling (LOO-PSIS; Vehtari et al. (2017)).

### S1.2 Prior specification

All fixed-effect coefficients on *v*, log *a*, and log *t* were given a weakly informative Normal(0, 0.25) prior on the link-appropriate scale (*v* on the natural scale; *a* and *t* on the log scale, so that a unit-magnitude coefficient corresponds to an exp(±0.49) ≈ ×1.6 multiplicative shift between cells). The Normal(0, 0.25) prior places 95% mass within ±0.49 on the link scale, which is mildly regularising but does not exclude the effect sizes typical of this paradigm. By-subject random-intercept standard deviations on *v*, *a*, and *t* were given a Weibull(α = 1.5, β = 0.3) hyperprior, which places 95% mass below ≈ 0.78 and is the HSSM 0.3.0 default for hierarchical SDs. Lapse contamination was handled with the standard HSSM mixture: a fixed 5% lapse probability with lapse trial reaction times drawn from Uniform(0, 20) seconds.

### S1.3 M3 posterior-predictive check and the v–a–t identifiability ridge

LOO-PSIS favours the full model M3, but with only Volatility (a single 2-level factor) entering both *a* and *t*, the joint posterior is subject to a known identifiability ridge in DDMs. Boundary inflation, drift-rate slowing, and non-decision-time inflation can each absorb similar mean-RT slowdowns at the cell level, so the joint magnitudes of *a*, *v*, and *t* are precise within the ridge but not uniquely partitioned. The fitted M3 posterior illustrates the ridge directly. It settles on an extreme parameter partition in which the regime contrast is carried jointly by a large boundary shift (*a*high/*a*low ≈ 2.80) and an opposing non-decision-time shift (*t*high ≈ 0.23 s vs. *t*low ≈ 0.72 s), whereas the boundary-only projection M2 expresses the same regime contrast as a modest *a*high/*a*low = 1.19. A posterior-predictive check shows that this extreme partition is nevertheless predictively adequate because forward-simulating full datasets from the joint M3 posterior (per-draw parameter sets with the fitted by-subject offsets; absorbing bounds at ±*a*) reproduces the observed RT distributions closely in all four Volatility × Distractor cells, with predicted cell means within 9–23 ms of the empirical values (S1 Fig). That two very different partitions of the same slowdown, M3’s boundary-plus-non-decision trade-off and M2’s modest boundary shift, are both compatible with the data at the predictive level is the practical signature of the ridge. The trial-level likelihood constrains the condition-level RT structure but not its unique division into drift, boundary, and non-decision components. We therefore use M3 only as evidence that the condition-level RT pattern is latent-decision relevant, and treat M1 and M2 as descriptive projections rather than as a uniquely identified decomposition of the mechanism.

### S1.4 Subject-conditional posterior-predictive checks of the marginal models

The conservative single-margin parameter-recovery test we ran (K = 20 trials/cell, a tenth of the 200 trials/cell in the fitted datasets and ~35× below the raw per-subject per-cell mean of ~700) was compromised both by small-N sampling conflation and by a simulator boundary-convention error identified in a subsequent audit (see Scope). A representative-N recovery refit was also infeasible within the available compute window because the no-RE HSSM smoke-fit attempt implied ≥ 6 hr per grid. We therefore use subject-conditional posterior-predictive checks (PPCs) as empirical-N directional diagnostics. The diagnostic question is whether the fitted M1 and M2 posteriors reproduce the direction of the empirical regime contrast at the sample size of the actual study, without the small-K identifiability conflation.

**Method.** We drew n = 20 posterior samples (without replacement) from each fitted M1 and M2 chain × draw posterior and, for each draw, simulated one full design-matched dataset (21 retained subjects × 2 volatilities × 2 distractor conditions × K = 200 trials/cell ≈ 16,800 trials per simulated dataset, the same per-cell count as the fitted datasets in S1.1). Subject random offsets on *v*, *a*, and *t* were taken **directly from the matching posterior draw** (subject-conditional PPC against the actual cohort) rather than re-sampled from σ. The Wiener random walk used σdiffusion = 1 and dt = 0.001 s, with the two absorbing bounds separated by 2*a* and starting point *z* · 2*a* — the HSSM parameterisation (S1.1), in which *a* is half the classic boundary separation. Pre-processing matched the empirical pipeline: RT ∈ [0.150, 4.000] s, correct trials only. Empirical comparison values are computed from the full filtered behavioural dataset; because the fitted subsample is a uniform random draw within cells, the two agree in expectation.

**Empirical observable.** The diagnostically informative observable is the trial-level (high − low) RT-interference regime contrast, defined as the per-subject (RTpresent − RTabsent) collapsed across same-location run position and then averaged within volatility. This contrast is invariant to absolute scale and tests whether either marginal DDM directionally reproduces the empirical sign at the trial-marginal level. This is not the rep-balanced interference contrast that the main text reports as its primary ANOVA. The rep-balanced average treats each run-position cell with equal weight and yields a (high − low) of −35.0 ms, since the rep-mix asymmetry between regimes flips the sign at the unfiltered level.

**Verdict.** The two rows of S1 Table must be read differently. M1 (drift-only) makes a genuine prediction for the contrast, because its drift rate varies across the four Volatility × Distractor cells. However, it misidentifies the direction. Its predictive median is −20 ms, and the empirical +15.4 ms lies outside the M1 90% interval. M2 (boundary-only) has no *v*, *a*, or *t* term that varies with Distractor, so it predicts an interference cost of essentially zero in both regimes (predictive medians of −1 and −6 ms, vs. empirical costs of +186 and +170 ms). Its tabled contrast distribution — median +8 ms, 90% interval [−19, +19] ms — is the sampling variation of the difference of those two near-zero costs across the 20 simulated datasets (mean +5 ± 12 ms, t(19) = 1.99, p = .061 against zero), so the interval width reflects simulation noise, not a modelled interference effect. The “Yes” in the M2 row therefore records only that the small empirical contrast falls inside that noise band (as any comparably small value would) and is not positive support for a boundary account. At the absolute level, both marginal models reproduce the empirical RT scale and accuracy well (e.g., M1 cell means within 5–48 ms of the empirical 0.97–1.29 s means; accuracies within ~2.5 pp), so the diagnostic burden falls on the structured contrasts rather than on overall scale.

Together with the S1.3 ridge argument, the PPC explains why the modelling identifies the decision architecture without uniquely partitioning it. The trial-level likelihood is sensitive to multiple latent-decision margins, but the present design does not split the high-volatility slowdown uniquely into drift, boundary, and non-decision-time components.

**Scope.** PPCs do not replace a strict frequentist parameter-recovery test. The conservative K = 20 single-margin recovery we ran earlier is no longer interpretable. An audit found that its data generator simulated with absorbing bounds at 0 and *a* (classic separation-*a* convention) while the HSSM likelihood used for refitting places the bounds at ±*a*, so the refitted boundary was systematically biased toward half the generating value (log-scale bias −0.45 to −0.46 across refits, vs. −0.69 for a pure halving, with the remainder absorbed by prior shrinkage and compensating drift and non-decision-time shifts). This mismatch is by itself sufficient to produce the 0% coverage previously observed on a_Intercept_, and it contaminates the remaining coverage figures, which we therefore do not report. A convention-consistent recovery refit at representative sample sizes awaits a longer compute window. The PPCs reported here therefore stand as empirical-N directional evidence on the regime contrast, not as a replacement for a strict refit-recovery.

### S1.5 Cell-level magnitudes and scope

The marginal models are directionally consistent with the behavioural reading that high-volatility blocks are associated with slower, more cautious responding. These magnitudes do not, however, evidence a present-trial gating impairment. In M1 the drift reduction under high volatility is larger on distractor-absent trials (Δ*v* = −0.52, 95% HDI [−0.59, −0.46]) than on distractor-present trials (−0.29 [−0.34, −0.24]), and the distractor-related drift cost (present minus absent) is smaller under high volatility (−0.46 [−0.51, −0.41]) than under low (−0.69 [−0.75, −0.63]). Because distractor-absent trials carry no same-location repetition mixture, the absent-trial estimate identifies the drift reduction as a per-trial effect of volatility on evidence accumulation in the manual decision. Present-trial estimates additionally inherit the repetition mixture that motivates the main-text reanalysis. The modelling therefore supports a latent-decision interpretation in which both evidence quality and response criterion shift under high volatility, while the stage-specific claim in the main text rests on eye-tracking and repetition-aware behavioural contrasts.

# References

Fengler, Alexander, Krishn Bera, Mads L. Pedersen, and Michael J. Frank. 2022. “Beyond Drift Diffusion Models: Fitting a Broad Class of Decision and Reinforcement Learning Models with HDDM.” Journal of Cognitive Neuroscience 34 (10): 1780–1805. https://doi.org/10.1162/jocn_a_01902.

Ratcliff, Roger, and Gail McKoon. 2008. “The Diffusion Decision Model: Theory and Data for Two-Choice Decision Tasks.” Neural Computation 20 (4): 873–922. https://doi.org/10.1162/neco.2008.12-06-420.

Vehtari, Aki, Andrew Gelman, and Jonah Gabry. 2017. “Practical Bayesian Model Evaluation Using Leave- One-Out Cross-Validation and WAIC.” Statistics and Computing 27 (5): 1413–32. https://doi.org/10.1007/s11222-016-9696-4.
