# Supplementary material for: Environmental volatility is associated with adaptive caution in visual search
Source: PLoS Comput Biol. 2026 Oct 21;22(10):e1014310. doi: 10.1371/journal.pcbi.1014310 (PMC13712733; doi:10.1371/journal.pcbi.1014310)
Supplement: Supplementary file 2 [file pcbi.1014310.s002.docx]

## S2 Text. Reduced VKF dynamic-state comparison

As a dynamic-state complement to the HSSM analysis, we fit reduced VKF + DDM-style RT models in which a trial-wise latent state π*t* tracking the participant’s running belief about distractor presence was updated by a Volatility Kalman Filter–like rule (Piray & Daw, 2021) and then linked to the drift rate, the boundary, both, or neither.

### S2.1 Latent volatility-tracking model

Let *x*_t_ ∈ {0, 1} indicate whether a distractor was present on trial *t*, *m*_t_ be the running estimate of distractor probability (initialised at *m*_0_ = 0.5), *ν*_t_ a running estimate of volatility (initialised at *ν*_0_ = 0.10), and π_t_ the trial-aligned latent state (initialised at π_0_, a free parameter). On each trial the prediction error *δ*_t_ = *x*_t_ − *m*_t−1_ drives a volatility-modulated learning rate

α_t_ = σ(η_0_ + η_1_ *ν*_t−1_),

with σ the logistic function and η_0_, η_1_ free parameters; the three latent states are then updated as

*m*_t_ = *m*_t−1_ + α_t_ *δ*_t_,

*ν*_t_ = (1 − ρ) *ν*_t−1_ + ρ |*δ*_t_|,

π_t_ = clip(π_t−1_ + α_t_ *δ*_t_, −3, +3),

with ρ ∈ (0, 1) a free volatility-update rate. Within each subject π_t_ was centred and z-scored before entering the decision-parameter equations below.

### S2.2 Candidate decision-parameter couplings

Trial-level drift rate *v*_t_ and boundary *a*_t_ were linked to π_t_ through one of four architectures. With *g*_t_ a present-trial gain indicator and *v*_0_, β_g_, *a*_0_, *w*_v_, *w*_a_ free parameters:

- **Null** (no latent-state coupling): *v*_t_ = *v*_0_·exp(β_g_ *g*_t_), *a*_t_ = *a*_0_.
- **Drift-state**  (latent state modulates drift): *v*_t_ = *v*_0_·exp(β_g_ *g*_t_) + *w*_v_ π_t_, *a*_t_ = *a*_0_.
- **Boundary-state** (latent state modulates boundary): *v*_t_ =*v*_0_·exp(β_g_ *g*_t_),*a*_t_=*a*_0_· exp(*w*_a_ π_t_).
- **Full** (latent state modulates both): *v*_t_ = *v*_0_·exp(β_g_ *g*_t_) + *w*_v_ π_t_, *a*_t_ = *a*_0_·exp(*w*_a_ π_t_).

The trial-level likelihood combined a Bernoulli term for response correctness with choice probability *p*(correct) = σ(2 *a*_t_ *v*_t_) and a Gaussian RT term around the analytic DDM mean *t*_0_ + (*a*_t_ / |*v*_t_|) tanh(*a*_t_ |*v*_t_|) with free residual SD σ_RT_. Models were fit subjectwise by maximum likelihood (multi-start L-BFGS-B with bounded parameters; four random starts; ≤ 800 iterations per start) and aggregated with AIC/BIC.

### S2.3 Sample, fit results, and scope

After RT filtering (0.150–4.000 s) and removal of missing core fields, the analysis included 60,255 trials from 21 subjects. AIC favoured the joint drift/boundary model, followed by the drift-state, gain-only, and boundary-state models:

| **Model** | **AIC** | **ΔAIC** | **BIC** | **ΔBIC** |
| --- | --- | --- | --- | --- |
| Full Model | 80245.84 | 0.00 | 82326.31 | 312.23 |
| Drift-state | 80562.11 | 316.26 | 82453.44 | 439.36 |
| Null | 81068.42 | 822.57 | 82014.08 | 0.00 |
| Boundary-state | 82061.02 | 1815.18 | 83952.35 | 1938.27 |

Subject-wise diagnostics showed that the full model improved negative log likelihood relative to the gain-only model for 21/21 subjects (median ΔNLL = −5.93), the drift-state model for 19/21 subjects (median ΔNLL = −4.15), and the boundary-state model for 16/21 subjects (median ΔNLL = −3.35). The boundary-only model remained the least stable of the dynamic architectures. Its worst subjectwise ΔNLL relative to the gain-only baseline was +322.60.

The VKF result is therefore compatible with the HSSM conclusion at the broad architectural level. AIC favours a model that allows latent state to influence both drift-like and boundary-like terms, whereas BIC favours the simpler gain-only baseline because the dynamic subjectwise models carry many additional parameters. The VKF likelihood is also a simplified RT-moment approximation. We therefore treat VKF as a supportive dynamic-state check, showing that a boundary-only account is insufficient for the manual RT data; the stage-specific attentional interpretation remains anchored in the repetition-aware eye-tracking analyses.

# References

Piray, Payam, and Nathaniel D. Daw. 2021. “A Model for Learning Based on the Joint Estimation of Stochas- ticity and Volatility.” Nature Communications 12(1): 6587. https://doi.org/10.1038/s41467-021-26731-9.
