# Supplementary material for: Environmental volatility is associated with adaptive caution in visual search
Source: PLoS Comput Biol. 2026 Oct 21;22(10):e1014310. doi: 10.1371/journal.pcbi.1014310 (PMC13712733; doi:10.1371/journal.pcbi.1014310)
Supplement: Supplementary file 3 [file pcbi.1014310.s003.docx]

## S3 Text. Post-capture dwell duration

Post-capture engagement was assessed on present trials where the first fixation landed on the distractor. First-distractor dwell duration did not reliably differ between high- and low-volatility blocks (185.9 vs 186.4 ms; mean difference -0.5 ms), *t*(20) = -0.06, *p* = .956. An equivalence test against the pre-specified +/-24.4 ms smallest effect size of interest was significant, TOST *p* = .0061, and the corresponding default JZS Bayes factor gave moderate support for the null, BF01 = 4.39. This result is evidence against a volatility effect on dwell at the stated bound, not a claim that post-capture dwell is generically identical across all possible effect sizes.
