# Supplementary material for: Environmental volatility is associated with adaptive caution in visual search
Source: PLoS Comput Biol. 2026 Oct 21;22(10):e1014310. doi: 10.1371/journal.pcbi.1014310 (PMC13712733; doi:10.1371/journal.pcbi.1014310)
Supplement: Supplementary file 4 [file pcbi.1014310.s004.docx]

## S4 Text. Present-trial first-saccade latency by same-location repetition

Oculomotor capture indexes *where* the first saccade lands. First-saccade latency indexes *when* it is launched. The two readouts can dissociate because volatility could in principle delay the first overt shift of gaze even if its destination is unchanged. We therefore analysed present-trial first-saccade latency with the same 2 (Volatility) × 3 (same-location run position) repeated-measures design used for oculomotor capture (*n* = 21; S2 Fig plots the measures in the format of Fig 3).

Latency showed no detectable volatility main effect, either across all first saccades, *F*(1, 20) = 1.39, *p* = .252, or when restricted to saccades landing on the distractor, *F*(1, 20) = 1.02, *p* = .324; nor was there a detectable Volatility × run-position interaction in either measure (*F*(2, 40) = 2.78, *p* = .074; *F*(2, 40) = 0.44, *p* = .648). Run position itself modulated overall latency, *F*(2, 40) = 5.55, *p* = .007. The two readouts of the same first saccade therefore converge. Neither where the first saccade lands nor when it is launched differed detectably across volatility regimes once same-location repetition was matched.

We note the limits of this null. A paired equivalence test on the collapsed latency contrast at a SESOI of *d*z = 0.5 (≈ 27 ms) was not significant (TOST *p* = .219), and the default JZS Bayes factor was only weakly informative (BF₀₁ = 1.66). The latency data therefore fail to detect a volatility effect but do not establish equivalence, and we do not treat them as positive evidence that first-saccade timing is unaffected.
