# Supplementary material for: Environmental volatility is associated with adaptive caution in visual search
Source: PLoS Comput Biol. 2026 Oct 21;22(10):e1014310. doi: 10.1371/journal.pcbi.1014310 (PMC13712733; doi:10.1371/journal.pcbi.1014310)
Supplement: Supplementary file 5 [file pcbi.1014310.s005.docx]

## S5 Text. Inter-trial switch costs in manual RT

As a complementary description of the absent-trial baseline shift, we computed two inter-trial switch costs from the manual RT record:

- **Appearance cost** (AP − PP) — the additional cost of a distractor that appears after an absent trial, relative to one that repeats from the previous (present) trial.
- **Lingering cost** (PA − AA) — the residual cost of an absent trial that follows a present trial, relative to a repeated absent trial.

Both contrasts are defined relative to the inter-trial baseline that the absent trial provides. Under the baseline-inflation account, both should be larger in the low-volatility regime (where the absent-trial baseline is lower) than in the high-volatility regime, because the baseline against which each switch cost is computed sits closer to the present-trial RT in the high-volatility regime.

A 2 (Volatility: high vs. low) × 2 (Switch-cost type: appearance vs. lingering) repeated-measures ANOVA on RT switch costs yielded a main effect of Volatility, *F*(1, 20) = 10.44, *p* = .004, ηp² = .34, with switch costs *larger* in low- than in high-volatility sessions (60.2 vs. 45.3 ms). This was in the same direction as the present − absent RT-defined distractor interference contrast. The main effect of Switch-cost type was strong, *F*(1, 20) = 149.60, *p* < .001, ηp² = .88, with appearance costs substantially larger than lingering costs (87.9 vs. 17.6 ms). The Volatility × Switch-cost type interaction was not reliable, *F*(1, 20) = 3.61, *p* = .072, ηp² = .15. The corresponding error-rate ANOVA yielded no reliable effects (all *p*s ≥ .093).

Both apparent reductions of RT-defined distractor interference under high volatility, the present − absent contrast tested in the main text and the appearance/lingering switch costs tested here, are consistent with the same descriptive source. This source is a higher absent-trial baseline under high volatility, rather than a detectable reduction in present-trial oculomotor capture after same-location repetition is matched.
