# Supplementary material for: Environmental volatility is associated with adaptive caution in visual search
Source: PLoS Comput Biol. 2026 Oct 21;22(10):e1014310. doi: 10.1371/journal.pcbi.1014310 (PMC13712733; doi:10.1371/journal.pcbi.1014310)
Supplement: Supplementary file 6 [file pcbi.1014310.s006.docx]

## S6 Text. Distractor-absent profiles: per-participant data and dependence among measures

*Dependence among the measures.* The five distractor-absent measures are not independent tests. Across-subject correlations of the high-minus-low differences show moderate coupling between total fixations and target latency (*r* = +0.47) and a speed–accuracy axis between error rate and final-target dwell (*r* = −0.41). The measures span roughly two underlying dimensions, a verification-prolongation axis and a speed–accuracy trade-off. The omnidirectional Hotelling’s T² reported in the main text is conservative under these conditions because it spreads power across all five dimensions and discounts the redundancy among positively correlated, co-directional measures, which is why the pre-specified directional composite is the matched test of the caution prediction.
