# Supplementary material for: Environmental volatility is associated with adaptive caution in visual search
Source: PLoS Comput Biol. 2026 Oct 21;22(10):e1014310. doi: 10.1371/journal.pcbi.1014310 (PMC13712733; doi:10.1371/journal.pcbi.1014310)
Supplement: Supplementary file 7 [file pcbi.1014310.s007.docx]

## S7 Text. Distractor-absent first-saccade allocation

We asked whether volatility altered *which* object the first saccade reached on distractor-absent trials and when it was launched. The proportion of first saccades that landed on the target did not differ reliably between regimes (56.0% vs. 52.1%; +3.9 pp), paired *t*(20) = 1.07, *p* = .295. Decomposed by destination, first-saccade latency did not differ across regimes overall (−7.9 ms, *t*(20) = −0.82, *p* = .424), nor for saccades reaching the target (−11.5 ms, *p* = .307) or a non-target placeholder (−0.8 ms, *p* = .928). The across-subject coupling between Δproportion-to-target and Δfirst-saccade latency was null (*r* = −0.05). Neither the destination nor the timing of the first saccade carried a volatility signature, locating the distractor-absent verification profile after the first overt shift of gaze rather than at it.
