# Supplementary material for: Environmental volatility is associated with adaptive caution in visual search
Source: PLoS Comput Biol. 2026 Oct 21;22(10):e1014310. doi: 10.1371/journal.pcbi.1014310 (PMC13712733; doi:10.1371/journal.pcbi.1014310)
Supplement: Supplementary file 8 [file pcbi.1014310.s008.docx]

## S8 Text. Task-set inertia: distractor-absent profile across absent-run positions

An alternative reading of the distractor-absent volatility profile is task-set inertia: the profile might reflect task set lingering from the preceding distractor-present trial rather than a sustained shift in caution. Because high volatility produces more present-to-absent transitions, task-set inertia predicts a *decaying* signature. The high-minus-low volatility gap should be largest on the first absent trial after a transition and shrink as the absent run continues. The trial-history variable ndist_rep counts consecutive distractor-absent trials and resets on every present trial, so its first level is exactly the post-transition trial the inertia account singles out. For each of the five primary distractor-absent measures, all taken from the recovered eye-tracking record so that run position is defined identically, we computed the high-minus-low gap at absent run positions 1, 2, and 3+ and the Volatility × run-position interaction from a 2 (Volatility) × 3 (absent run position) repeated-measures ANOVA (*n* = 21).

The predicted decay is absent (S2 Table; Fig 4B in the main text). The gap at the first post-transition trial was no larger than at later positions for four of the five measures, and no measure showed a reliable Volatility × run-position interaction. The decay contrast, the gap at run position 1 minus the gap at run position 3+, is close to zero for RT, error rate, total fixations, and final-target dwell, and negative for target latency (the gap grew across the run). Within the same 2 × 3 models, the Volatility main effect held for total fixations and error rate and was marginal for final-target dwell. Under a first-order Markov process, the probability of a switch back to the distractor-present state is identical at every absent run position, so an observer who tracks the transition probability should show a flat, elevated profile; a carried-over task set should decay.

*Sensitivity.* With *n* = 21 (df = 2, 40) and α = .05, the Volatility × run-position interaction has 80% power for effects of ηp² ≥ .206 (Cohen’s *f* ≥ 0.51; noncentrality λ = 10.39). The observed interactions ranged from ηp² = .009 to .079. These tests therefore bound a decaying component and do not exclude it. Analysis script: analysis/code/revision2_absent_run_position.py.
