# Supplementary material for: Environmental volatility is associated with adaptive caution in visual search
Source: PLoS Comput Biol. 2026 Oct 21;22(10):e1014310. doi: 10.1371/journal.pcbi.1014310 (PMC13712733; doi:10.1371/journal.pcbi.1014310)
Supplement: Supplementary file 9 [file pcbi.1014310.s012.docx]

**S1 Table.** Subject-conditional posterior-predictive check of the marginal models M1 and M2 (*n* = 20 simulated full-design datasets per model). *Empirical* is the observed trial-pooled (high − low) difference in RT-defined distractor interference (RTpresent − RTabsent); it is identical for both rows because it is a property of the data, not the model. Predictive medians and intervals are quantiles across the 20 simulated datasets.

| **Model** | **Empirical** | **Predictive median** | **50% interval** | **90% interval** | **Empirical inside 90% PI?** |
| --- | --- | --- | --- | --- | --- |
| M1 (drift-only) | +15.4 ms | −20 ms | [−30, −6] | [−49, 2] | No (wrong sign) |
| M2 (boundary-only) | +15.4 ms | +8 ms | [+2, +15] | [−19, +19] | Yes (but see Verdict) |
