# Supplementary material for: Environmental volatility is associated with adaptive caution in visual search
Source: PLoS Comput Biol. 2026 Oct 21;22(10):e1014310. doi: 10.1371/journal.pcbi.1014310 (PMC13712733; doi:10.1371/journal.pcbi.1014310)
Supplement: Supplementary file 10 [file pcbi.1014310.s013.docx]

**S2 Table.** Distractor-absent volatility gap (high − low) at absent run positions 1, 2, and 3+; the Volatility main effect and the Volatility × run-position interaction from 2 × 3 repeated-measures ANOVAs; and the decay contrast, the gap at position 1 minus the gap at position 3+, in native units and in *d*z units (paired *t*-test; *n* = 21). A carried-over task set predicts a positive decay contrast for RT, target latency, total fixations, and final-target dwell, and a negative one for error rate.

| Measure | Pos. 1 | Pos. 2 | Pos. 3+ | Volatility, *F*(1, 20) | Interaction, *F*(2, 40) | Decay [95% CI] | Decay, *d*z [95% CI] | *t*(20) |
| --- | --- | --- | --- | --- | --- | --- | --- | --- |
| Reaction time (ms) | +117.9 | +137.4 | +117.0 | 2.45, *p* = .133 | 1.51, *p* = .232, ηp² = .070 | +1.0 [−27.6, +29.5] | +0.02 [−0.44, +0.47] | 0.07, *p* = .945 |
| Error rate (pp) | −0.71 | −0.99 | −0.85 | 4.35, *p* = .050 | 0.17, *p* = .841, ηp² = .009 | +0.13 [−1.05, +1.32] | +0.05 [−0.40, +0.51] | 0.23, *p* = .820 |
| Target latency (ms) | −8.2 | +4.9 | +6.7 | 0.00, *p* = .947 | 1.71, *p* = .193, ηp² = .079 | −14.9 [−29.0, −0.7] | −0.48 [−0.93, −0.02] | −2.19, *p* = .041 |
| Total fixations | +0.26 | +0.35 | +0.29 | 5.08, *p* = .036 | 1.68, *p* = .199, ηp² = .078 | −0.02 [−0.12, +0.07] | −0.11 [−0.57, +0.34] | −0.52, *p* = .609 |
| Final-target dwell (ms) | +81.1 | +66.4 | +77.0 | 3.64, *p* = .071 | 0.67, *p* = .516, ηp² = .033 | +4.1 [−19.8, +28.0] | +0.08 [−0.38, +0.53] | 0.36, *p* = .726 |
